# Supplementary material for: Modeling glioblastoma heterogeneity as a dynamic network of cell states
Source: Mol Syst Biol. 2021 Sep 16;17(9):e10105. doi: 10.15252/msb.202010105 (PMC8444284; doi:10.15252/msb.202010105)
Supplement: Supplementary file 6 — Source Data for Figure 5 [file MSB-17-e10105-s004.zip › Figure5A_sourcedata/GSEA_3017/hallmarks_stateC.GseaPreranked.1621934520804/HALLMARK_ANDROGEN_RESPONSE.html]

Details for gene set HALLMARK\_ANDROGEN\_RESPONSE[GSEA]

|  || Dataset | state13017 |
| Phenotype | NoPhenotypeAvailable |
| Upregulated in class | na\_neg |
| GeneSet | HALLMARK\_ANDROGEN\_RESPONSE |
| Enrichment Score (ES) | -0.3255241 |
| Normalized Enrichment Score (NES) | -1.2614962 |
| Nominal p-value | 0.20785597 |
| FDR q-value | 0.21107927 |
| FWER p-Value | 0.924 |
Table: GSEA Results Summary

  

Fig 1: Enrichment plot: HALLMARK\_ANDROGEN\_RESPONSE      
 Profile of the Running ES Score & Positions of GeneSet Members on the Rank Ordered List

  

| PROBE | GENE SYMBOL | GENE\_TITLE | RANK IN GENE LIST | RANK METRIC SCORE | RUNNING ES | CORE ENRICHMENT || 1 | CDK6 |  |  | 24 | 0.518 | 0.0505 | No |
| 2 | XRCC5 |  |  | 188 | 0.259 | -0.1841 | No |
| 3 | MAF |  |  | 196 | -0.251 | -0.1518 | No |
| 4 | SAT1 |  |  | 241 | -0.262 | -0.1810 | No |
| 5 | ABHD2 |  |  | 296 | -0.275 | -0.2253 | No |
| 6 | LIFR |  |  | 311 | -0.282 | -0.1994 | No |
| 7 | PMEPA1 |  |  | 375 | -0.308 | -0.2532 | No |
| 8 | HMGCR |  |  | 418 | -0.334 | -0.2663 | Yes |
| 9 | INSIG1 |  |  | 434 | -0.342 | -0.2315 | Yes |
| 10 | MYL12A |  |  | 448 | -0.350 | -0.1917 | Yes |
| 11 | IDI1 |  |  | 451 | -0.353 | -0.1327 | Yes |
| 12 | B2M |  |  | 468 | -0.371 | -0.0945 | Yes |
| 13 | TSC22D1 |  |  | 492 | -0.400 | -0.0632 | Yes |
| 14 | SCD |  |  | 504 | -0.431 | -0.0057 | Yes |
| 15 | DBI |  |  | 514 | -0.440 | 0.0568 | Yes |
| 16 | HMGCS1 |  |  | 529 | -0.466 | 0.1153 | Yes |
Table: GSEA details [plain text format]

  

Fig 2: HALLMARK\_ANDROGEN\_RESPONSE: Random ES distribution      
 Gene set null distribution of ES for **HALLMARK\_ANDROGEN\_RESPONSE**
